# Antimicrobial Face Shield: Next Generation of Facial Protective Equipment against SARS-CoV-2 and Multidrug-Resistant Bacteria

**DOI:** 10.3390/ijms22179518

**Published:** 2021-09-01

**Authors:** Alberto Tuñón-Molina, Miguel Martí, Yukiko Muramoto, Takeshi Noda, Kazuo Takayama, Ángel Serrano-Aroca

**Affiliations:** 1 Biomaterials and Bioengineering Lab., Centro de Investigación Traslacional San Alberto Magno, Universidad Católica de Valencia San Vicente Mártir, c/Guillem de Castro 94, 46001 Valencia, Spain; alberto.tunon@ucv.es (A.T.-M.); miguel.marti@ucv.es (M.M.); 2 Laboratory of Ultrastructural Virology, Institute for Frontier Life and Medical Sciences, Kyoto University, Kyoto 606-8507, Japan; muramo@infront.kyoto-u.ac.jp (Y.M.); t-noda@infront.kyoto-u.ac.jp (T.N.); 3 Center for iPS Cell Research and Application (CiRA), Kyoto University, Kyoto 606-8507, Japan

**Keywords:** face shield, facial protective equipment, SARS-CoV-2, phage phi 6, MRSA, MRSE, polyethylene terephthalate, benzalkonium chloride, COVID-19, multidrug-resistant bacteria

## Abstract

Transparent materials used for facial protection equipment provide protection against microbial infections caused by viruses and bacteria, including multidrug-resistant strains. However, transparent materials used for this type of application are made of materials that do not possess antimicrobial activity. They just avoid direct contact between the person and the biological agent. Therefore, healthy people can become infected through contact of the contaminated material surfaces and this equipment constitute an increasing source of infectious biological waste. Furthermore, infected people can transmit microbial infections easily because the protective equipment do not inactivate the microbial load generated while breathing, sneezing or coughing. In this regard, the goal of this work consisted of fabricating a transparent face shield with intrinsic antimicrobial activity that could provide extra-protection against infectious agents and reduce the generation of infectious waste. Thus, a single-use transparent antimicrobial face shield composed of polyethylene terephthalate and an antimicrobial coating of benzalkonium chloride has been developed for the next generation of facial protective equipment. The antimicrobial coating was analyzed by atomic force microscopy and field emission scanning electron microscopy with elemental analysis. This is the first facial transparent protective material capable of inactivating enveloped viruses such as severe acute respiratory syndrome coronavirus 2 (SARS-CoV-2) in less than one minute of contact, and the methicillin-resistant *Staphylococcus aureus* and *Staphylococcus epidermidis*. Bacterial infections contribute to severe pneumonia associated with the SARS-CoV-2 infection, and their resistance to antibiotics is increasing. Our extra protective broad-spectrum antimicrobial composite material could also be applied for the fabrication of other facial protective tools such as such as goggles, helmets, plastic masks and space separation screens used for counters or vehicles. This low-cost technology would be very useful to combat the current pandemic and protect health care workers from multidrug-resistant infections in developed and underdeveloped countries.

## 1. Introduction

Even though the severe lockdowns carried out in many countries of the world, the coronavirus disease 2019 (COVID-19) pandemic is still increasing the number of global deaths in most countries [1,2,3]. The causative agent of this disease is the severe acute respiratory syndrome coronavirus 2 (SARS-CoV-2), which is an enveloped positive-sense single-stranded RNA virus [4] that belongs to the IV Baltimore group [5]. SARS-CoV-2 causes atypical viral pneumonia [6,7] which death risk increases by co-infection with bacteria such as *Streptococcus pneumoniae* [8,9,10,11].

The emergence of highly pathogenic viruses, such as SARS-CoV-2, that can co-infect with other viruses or bacteria [12], including antibiotic-resistant strains, constitutes one of the most current threatening to humans in this century. Additionally, bacterial resistance to antibiotics in pneumonia treatment is increasing at an alarming rate [13,14]. SARS-CoV-2 showed high stability in different material surfaces, including the surface of metals, plastics and cardboard [15,16,17,18,19]. Therefore, in addition to the aerosol transmission route, SARS-CoV-2 can be transmitted by contact with material surfaces contaminated with this pathogen [15,16,17,18,19,20]. In fact, it can spread faster than its two ancestors SARS-CoV and Middle East respiratory syndrome coronavirus [21] through coughing, sneezing, touching or breathing [22] and more easily through asymptomatic carriers [23,24].

Influenza virus (IFV) affects the nasal mucosa in the course of infections that simultaneously affect other sectors of the respiratory tract, including the lower tract [25]. IFV is also an enveloped single-stranded RNA virus-like SARS-CoV-2 [26]. Another important global risk is caused by respiratory infections caused by bacteria such as *S. pneumoniae* that is the most frequently isolated organism with the highest mortality [27]. This pathogen is the cause of many respiratory processes such as pneumonia, otitis, sinusitis, complicated with sepsis, meningitis and abscesses [28,29]. Apart from the therapeutic therapies aimed at combating these diseases and in those cases in which there are no effective therapies for the treatment of the infections caused by these pathogens, facial protection equipment acquires great importance. Facial protection equipment against infectious biological agents includes those with eye and/or respiratory protection (nose and mouth) to prevent the entry of microorganisms, splashes and biological aerosols through the respiratory or mucous tract.

The choice of a specific type of protection resides in the choice of equipment according to its application. Thus, there is protective equipment such as face masks that are made by porous fabric that filtrates the air and impede the pass of most of the microbial particles [30]. Another option of protective equipment is commonly called as face shields made of transparent plastic materials [31]. This type of protective equipment acts by forming a barrier between the wearer of the screen and the biological agent, thus avoiding, in the best of cases, the entry of the agent through the respiratory and mucosal tracts. Even though its effectiveness in combination with other protection measures is not questioned, by itself, this type of protection is not totally effective as many of the infectious biological agents are capable of surviving on its surface for a long time. Even though all the devices developed to date fulfill the function of acting as a barrier against direct exposure of the infectious biological agent, they may not be entirely effective, since the device has not been fabricated with antimicrobial materials capable of inactivating infectious agents when they are in contact with the material surface. Furthermore, this contaminated biological waste constitutes an environmental risk associated with the waste management of these protective systems.

Polyethylene terephthalate (PET) is a commercial low-cost transparent and recyclable polyester that is commonly used for the fabrication of facial protective equipment such as face shields [32]. However, this plastic material does not possess antimicrobial properties.

In this regard, quaternary ammonium compounds such as benzalkonium chloride (BAK) have been confirmed to be capable of inactivating enveloped RNA viruses [33] and Gram-positive multidrug-resistant bacteria [34]. In fact, this chemical compound is widely used as a disinfectant against bacteria, viruses, pathogenic fungi and mycobacteria [35].

The goal of this work consisted of producing a transparent face shield capable of providing extra-protection by acting as a physic barrier with intrinsic antimicrobial activity against enveloped viruses such as SARS-CoV-2 and bacteriophage phi 6, and multidrug-resistant bacteria.

## 2. Results and Discussion

### 2.1. Composite Material Morphology

**Figure 1:**
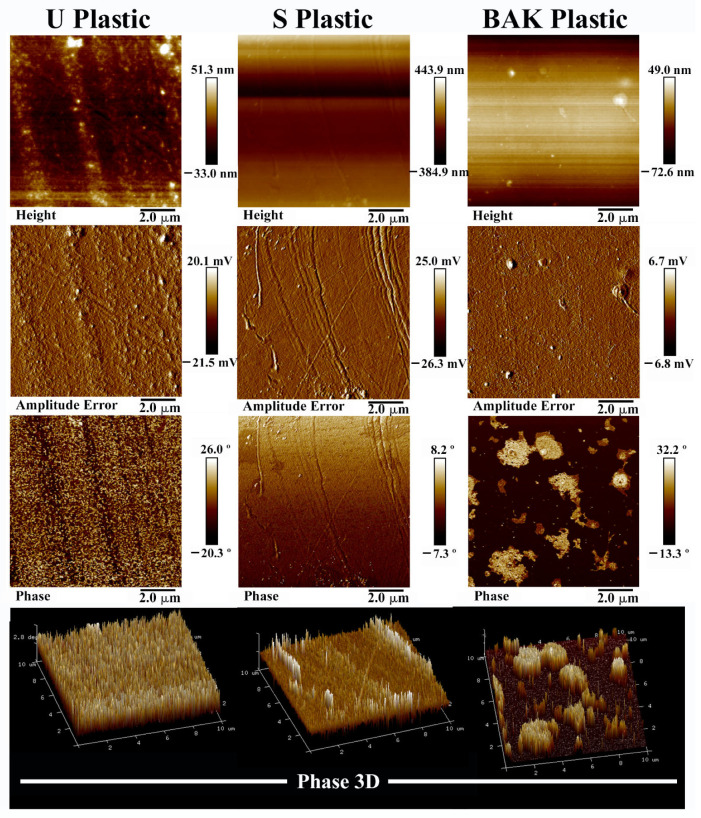
AFM topography images (height, amplitude error and phase images) and phase 3D representation recorded in tapping mode of the Untreated plastic (U Plastic), plastic treated by dip coating with the ethanol-based solvent (S Plastic) and filter with the biofunctional benzalkonium chloride (BAK) coating (BAK Plastic) scanning a 10 µm × 10 µm area. These images show how the BAK coating was formed on the polyethylene terephthalate surface after the dip-coating treatment. The U Plastic shows some impurities on its surface which disappear after immersion in ethanol (S Plastic). Thus, the clean S Plastic shows some surface imperfections (irregular stripes) which disappear after the BAK coating (BAK Plastic). The BAK plastic shows slightly higher zones observed as white zones in the phase image.

Atomic force microscopy (AFM) and field emission scanning electron microscope (FESEM) with elemental analysis were performed in order to characterize the BAK microcoating formed onto the PET surface. Figure 1 shows the AFM images of the treated and untreated PET plastics over a scan area of 10 µm × 10 µm.

**Figure 2:**
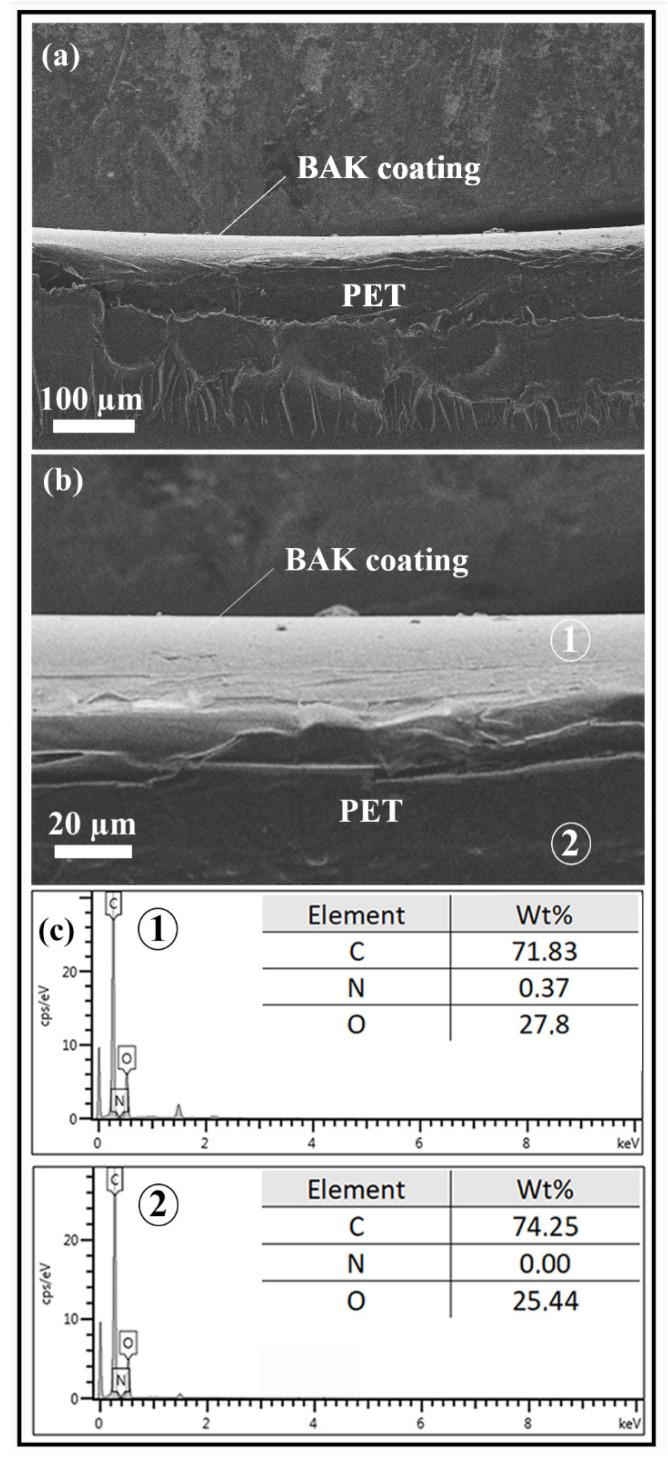
Morphology of the biofunctional coating of BAK onto the PET surface by Field Emission Scanning Electron Microscopy with energy-disperse X-ray spectroscopy (EDS) for elemental analysis: PET with 0.182 ± 0.034% *w/w* of biofunctional BAK coating (BAK Plastic) at two magnifications: (**a**) ×150 and (**b**) ×720, and (**c**) EDS elemental analysis of the coating and PET matrix.

The 2D phase provides images whose contrast is produced by differences in the adhesion and viscoelastic properties of the sample surface [36]. Thus, the pictures of the topography and phase angle clearly indicated that a BAK coating was formed onto the PET surface (BAK Plastic) after the dip-coating treatment with the solvent containing BAK (Figure 1). It can be clearly observed that the untreated plastic (U Plastic) possesses some impurities on its surface which disappear after immersing the disk in ethanol 70% for 1 min. Thus, the surface imperfections produced by the plastic fabrication procedure can be clearly observed in the 2D phase image of the PET plastic treated by dip coating with the absolute ethanol/distilled water (70/30 *v/v*) solvent mixture (S Plastic). However, the AFM images of the BAK Plastic clearly show that a BAK coating was formed covering all the imperfections observed in the 2D phase image of the S Plastic sample. This coating presents slightly higher zones that are observed white zones in the phase images. These results are in good agreement with the FESEM images shown in the following Figure 2.

The FESEM micrographs show clearly how a microcoating of BAK (light grey phase) is formed onto the PET surface (dark grey phase) with a thickness of approximately 25 µm (see Figure 2). Furthermore, the energy-disperse X-ray spectroscopy (EDS) analysis shows a nitrogen content of 0.37% weight on the BAK coating in good agreement with the nitrogen atom present in the BAK compound [34]. However, the EDS analysis on the PET matrix (polymer without nitrogen atoms) does not show any nitrogen content as expected.

### 2.2. Opacity

**Figure 3:**
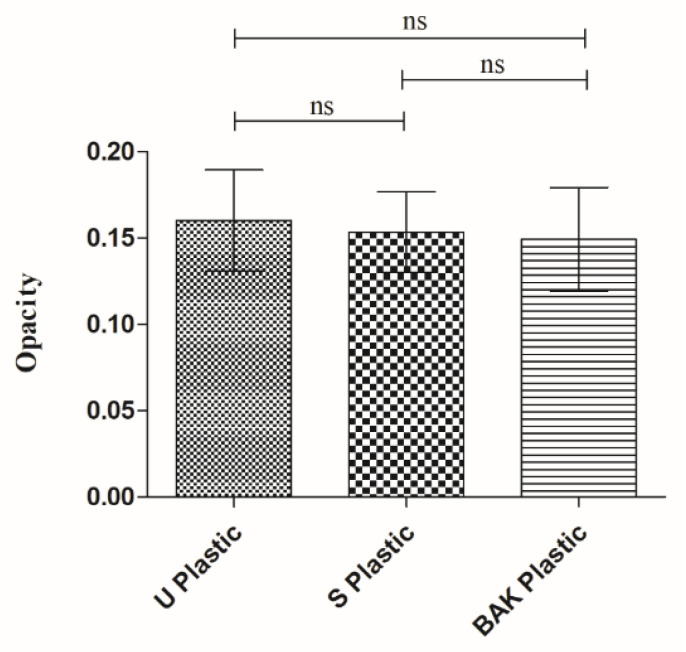
Opacity results of the Untreated PET (U Plastic), PET treated by dip coating with the ethanol-based solvent (S Plastic) and PET with the biofunctional BAK coating (BAK Plastic). The ANOVA results are indicated in this plot; ns: not significant.

Figure 3 shows that there are no statistically significant differences of opacity (or transparency), calculated with Equation (1), of the PET disks before and after the treatments with solvent or the dip-coating treatment with BAK, which is essential to be used as transparent facial protective equipment (face shield screens, plastic masks, protective screens, protective glasses, etc.).

### 2.3. Antibacterial Activity

**Figure 4:**
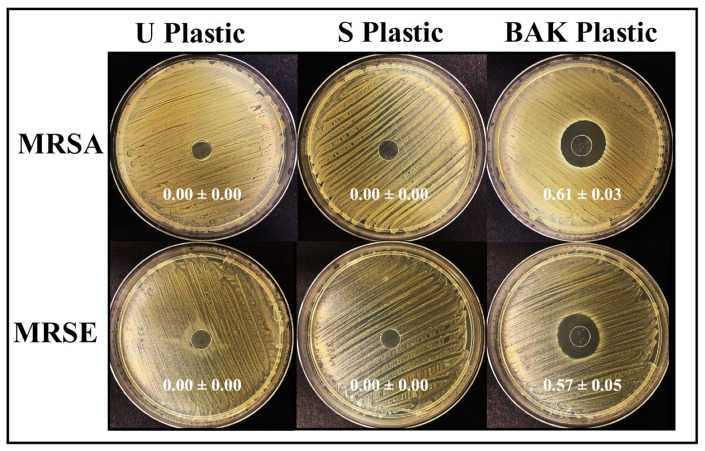
Antibacterial agar disk diffusion tests with two multidrug-resistant bacteria: methicillin-resistant *Staphylococcus aureus* (MRSA) and methicillin-resistant *Staphylococcus epidermidis* (MRSE). Untreated PET (U Plastic), PET treated by dip coating with the ethanol-based solvent (S Plastic) and the PET with the biofunctional BAK coating (BAK Plastic) after 24 h of incubation at 37 °C. The normalized widths of the antibacterial halos, expressed as mean ± standard deviation and calculated with equation (1), are shown in each image.

Figure 4 shows the antibacterial results achieved with the U Plastic, the S Plastic and the BAK Plastic against two multidrug-resistant bacteria: methicillin-resistant *Staphylococcus aureus* (MRSA) and methicillin-resistant *Staphylococcus epidermidis* (MRSE).

Therefore, we can observe that the plastic with the biofunctional coating of BAK showed potent antibacterial activity against MRSA and MRSE with similar normalized antibacterial halos of 0.61 ± 0.03 and 0.57 ± 0.05, respectively.

### 2.4. Antiviral Activity

The phage phi 6, which is an enveloped double-stranded RNA virus (group III of the Baltimore classification [5]), was used as biosafe viral model of SARS-CoV-2 and other enveloped viruses such as influenza due to safety reasons.

**Figure 5:**
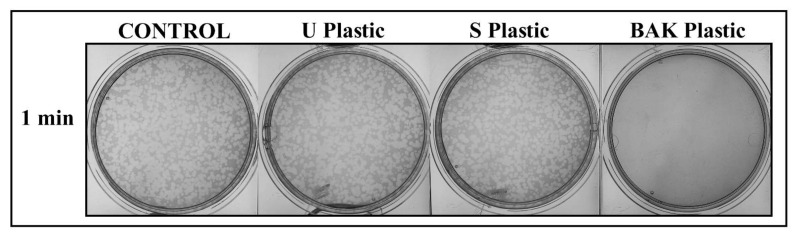
Loss of phage phi 6 viability measured by the double-layer method. Phage phi 6 titration images of undiluted samples for control, untreated PET (U Plastic), PET treated by dip coating with the ethanol-based solvent (S plastic) and PET with the biofunctional BAK coating (BAK plastic) at 1 min of viral contact.

The BAK Plastic showed potent antiviral activity against phage phi 6 (100% of viral inhibition, see Figure 5).

Thus, no plaques were produced on the bacterial lawns after 1 min of contact between the BAK Plastic and the biosafe viral model. However, similar plaques to control can be observed on the bacterial lawns after 1 min of contact between the U Plastic or S Plastic and the biosafe viral model (see Figure 5). The phage titers of each type of sample were calculated and compared with the control (Table 1).

**Figure 6:**
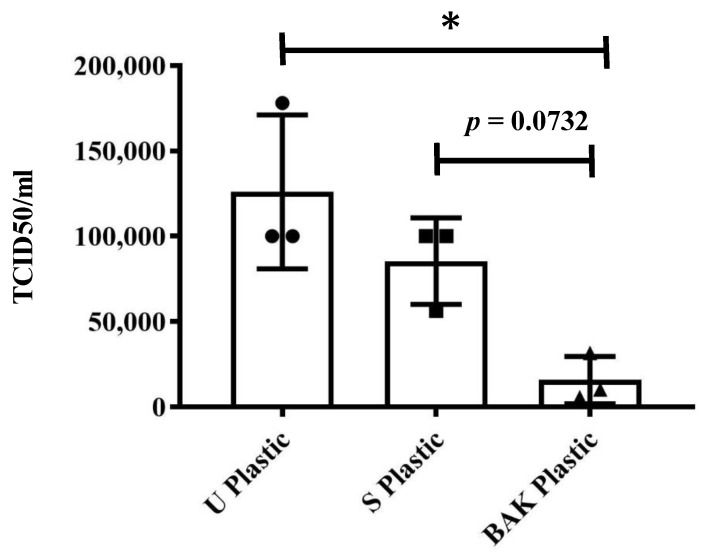
Reduction of infectious titers in PFU/mL of severe acute respiratory syndrome coronavirus 2 (SARS-CoV-2) after 1 min of contact determined by the median tissue culture infectious dose per mL (TCID50/mL) method. Untreated PET (U plastic), PET treated with the ethanol solvent (S Plastic) and the PET with the biofunctional BAK coating (BAK Plastic). A dot, square and triangle plot is a data set based on the value of each point. * *p* < 0.05.

Table 1 shows that the titers obtained by contacting the phages with the U or S Plastic are similar to the control. However, the BAK plastic displayed a strong phage inactivation. The results achieved with SARS-CoV-2 after 1 min of contact with the U Plastic, the S Plastic and the BAK Plastic containing the biofunctional coating are shown in Figure 6.

These results clearly demonstrate that the BAK Plastic is very effective against SARS-CoV-2 even after 1 min of contact. This is also in good agreement with the antiviral results of the biosafe viral model used in this study (see Figure 5 and Table 1). However, since BAK is highly water-soluble and therefore could come out when the PET sheet with the BAK coating is in contact with water, the antiviral and antibacterial tests were performed again after washing with distilled water to analyze the antimicrobial durability of the BAK coating to water. The results of these experiments (results not shown) showed that the antimicrobial BAK coating dissolves really fast in distilled water and loses its antimicrobial activity as expected. However, the developed antimicrobial face shield is presented here as a single-use face protective equipment for the current and future microbial menaces.

**Figure 7:**
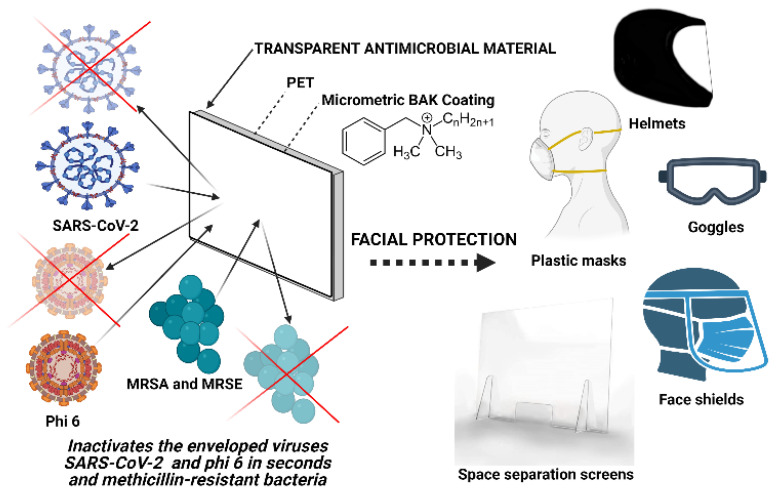
Applications of the coating technology of transparent polyethylene terephthalate (PET) with an antimicrobial coating of benzalkonium chloride (BAK) for the next generation of facial protective equipment: face shields, plastic masks, helmets, goggles, helmets and space separation screens.

These advanced face shields can provide superior protection to virologists working with highly infective pathogens in high-level biosafety labs, surgeons and healthcare workers in general. Furthermore, the proposed PET plastic is a recyclable material that can be reutilized [32] and thus contribute to decrease the increasing amount of this type of waste generated in the current pandemic. Furthermore, the antimicrobial coating can be produced easily as many times as necessary as a reusability characteristic of this technology. The BAK coating can be performed onto the outside side only for people not infected or also on the inside side if the protective equipment is going to be used for infected patients. It could also be performed on other types of transparent synthetic polymers such as polycarbonate, polymethyl methacrylate, poly (2-hydroxyethyl methacrylate), on non-transparent materials or on biodegradable polymers that would provide a solution to the need for bio-based protective tools for environmental reasons [37]. This next generation equipment will significantly reduce the increasing generation of infectious biological waste. These new technologies could revolutionize the face protective tool industry because other face protective equipment could be developed applying the same low-cost technology providing high antimicrobial activity (see Figure 7).

The antimicrobial mechanism of action of BAK against both bacterial and enveloped viruses is attributed to its positively charged nitrogen atoms that can eradicate the bacterial surface or disrupt the phospholipid bilayer membrane, the glycoproteinaceous envelope, and the spike glycoproteins of viruses such as phi6, SARS-CoV-2 and IFV [38,39]. BAK is a Food and Drug Administration-approved product for a broad-range of disinfecting applications such as additives in soaps and hand sanitizers [40,41,42]. We have demonstrated here that these transparent PET-based composites possess high antiviral and antibacterial activity to reduce the spread of COVID-19 and methicillin-resistant bacteria. This extra-protective composite material has been developed by a low-cost method of dip coating that let BAK to physically adsorbed [43] onto the surface of a commercial PET plastic commonly used for the fabrication of face shields and other protective equipment providing high antimicrobial activity. Nonetheless, further research is necessary to overcome the possible shortfalls on the applicability of this coating technology to other protective tools and to other types of transparent materials. The effect of packing, storage and transportation on the antimicrobial properties of the BAK coating need also to be studied before applying this technology at large-scale.

## 3. Materials and Methods

### 3.1. Dip Coating Treatment with Benzalkonium Chloride

Sheets of PET with a thickness of 0.3167 ± 0.0408 mm used for the fabrication of commercial face shields were purchased from Plasticos Villamarchante S.L [44] (Valencia, Spain). Six disk specimens (n = 6) of approximately 10 mm in diameter of these transparent PET sheets (BAK Plastic) were treated with 70% ethyl alcohol with 0.1% *w/w* BAK (Montplet, Barcelona, Spain) by the dip-coating method [45] for 1 min at 25 °C to achieve a dry BAK content of 0.182 ± 0.034% *w/w* determined gravimetrically. Six more PET disks (n = 6) were subjected to the same dip-coating treatment but using only an absolute ethanol/distilled water solution (70/30% *v/v*) without BAK for 1 min at 25 °C (S Plastic). Untreated PET disks (U Plastic) disks (n = 6) were produced as reference material. The disks were subsequently dried at 60 °C for 48 h and sterilized under UV radiation for 1 h per disk side. The BAK used in this study was previously characterized by nuclear magnetic resonance on a BRUKER AVIIIHD 800 MHz (Bruker BioSpin AG, Fälladen, Switzerland) equipped with a 5 mm cryogenic CP-TCI [34].

### 3.2. Atomic Force Microscopy

Atomic force microscopy (AFM) was performed with a Bruker MultiMode 8 scanning probe microscope (SPM, Karlsruhe, Germany) operating in tapping mode in air and with the NanoScope V Controller and NanoScope 8.15 software version (Bruker, Karlsruhe, Germany). An antimony (n) doped silicon cantilever from Bruker was used with a scan rate of 0.500 Hz. The phase signal was set to zero at the resonance frequency of the tip. The tapping frequency was 5–10% lower than the resonance frequency. The drive amplitude and amplitude setpoints were 308.5 and 644.8 mV, respectively, and the aspect ratio was 1.00.

### 3.3. Electron Microscopy

A Zeiss Ultra 55 field emission scanning electron microscope (FESEM, Jena, Germany) was operated at an accelerating voltage of 10 kV to observe the biofunctional coating morphology of the treated PET surface at a magnification of ×150 and ×720. The plastic samples were prepared to be conductive by platinum coating with a sputter coating unit. This FESEM microscope is equipped with energy-disperse X-ray spectroscopy (EDS) for elemental ratio estimation at 2.00 kV.

### 3.4. Opacity

The opacity of the synthesized films was evaluated according to the spectrophotometric method utilized by Park and Zhao [46]. Thus, rectangular specimens (4 mm × 50 mm) of the dry films were directly placed in a spectrophotometer cell to measure the absorbance at 600 nm with a UV/VIS Nanocolor UV0245 spectrophotometer (Macherey-Nagel, Düren, Germany). An empty cell was utilized as a reference. After that, the opacity (O) of the films can be determined with Equation (1), in which Abs600 is the absorbance value at 600 nm and x is film thickness in mm.

(1) $$O\;=\;\frac{Abs600}{x}$$

The measurements were performed with three specimens of each material and were reported as absorbance divided by film thickness (mean ± standard deviation).

### 3.5. Phage Host Culture

The phage phi 6 host is *Pseudomonas syringae* (DSM 21482). This Gram-negative bacterium was purchased from the Leibniz Institute DSMZ–German Collection of Microorganisms and Cell cultures GmbH (Braunschweig, Germany). *P. syringae* was cultured in solid tryptic soy agar (TSA, Liofilchem, Teramo, Italy). After that, the microorganism was cultured in liquid tryptic soy broth (TSB, Liofilchem, Roseto degli Abruzzi, Italy) incubated in an orbital shaker (CERTOMAT IS, Sartorius Stedim Biotech, Göttingen, Germany) at 25 °C and 120 rpm.

### 3.6. Phage Propagation

The specifications provided by the Leibniz Institute DSMZ–German Collection of Microorganisms and Cell Cultures GmbH were followed to propagate the phage phi 6 (DSM 21518) by using the double agar layer technique with top and bottom agar, which allows to perform lysis in bacterial host cultures [47].

### 3.7. Antiviral Test with the Biosafe Viral Model

50 μL of TSB with phages was placed onto each material disk at a titer of about 1 × 10^6^ plaque-forming units per mL (PFU/mL) and allowed to incubate for 1 min. Each material disk was placed in a falcon tube with 10 mL TSB, and subsequently sonicated for 5 min and vortexed for 1 min at room temperature (24 ± 1 °C).

Phage titration was performed by serial dilutions of each falcon sample. 100 μL of each phage dilution was mixed with 100 μL of the host strain at OD_600 nm_ = 0.5.

The infective activity of the phage phi 6 was measured based on the double-layer method [48]. Thus, 4 mL of top agar (TSB + 0.75% bacteriological agar, Scharlau) and 5 mM CaCl_2_ were added to the mixture containing phages and bacteria, which was then poured on TSA plates. Incubation of the plates was performed for 24–48 h in a refrigerated oven at 25 °C. Phage titers of each sample were calculated in PFU/mL and compared with a control consisting of 50 μL of phage added directly to the bacterial culture without being in contact with any type of disk and without sonication or vortexing.

The antiviral activity of the material disks was estimated at 1 min of contact with the biosafe virus model in log reductions of titers. It was ensured that the residual disinfectants present in the titrated samples did not interfere with the titration process. It was also checked that sonication and vortexing did not affect the infectious activity of the phage phi 6. The antiviral assays were carried out three times during two different days (n = 6) to ensure reproducible results.

### 3.8. Antiviral Tests Using SARS-CoV-2

The SARS-CoV-2 strain (SARS-CoV-2/Hu/DP/Kng/19-027) was provided to us by Dr. Tomohiko Takasaki and Dr. Jun-Ichi Sakuragi from the Kanagawa Prefectural Institute of Public Health. SARS-CoV-2 was plaque-purified, propagated in Vero cells and stored at −80 °C. 50 μL of a virus suspension in phosphate-buffered saline (PBS) was placed onto each material disk at a titer of 1.3 × 10^5^ median tissue culture infectious dose (TCID50) per disk, and then incubated for 1 min of contact at ambient temperature. After that, 1 mL PBS was added to each disk, and then vortexed for 5 min. After that, each tube was vortexed for 5 min at ambient temperature. Viral titers were determined by the TCID50 assay performed in a Biosafety Level 3 laboratory at Kyoto University. Thus, TMPRSS2/Vero cells [49] (JCRB1818, JCRB Cell Bank), cultured with the minimum essential media (MEM, Sigma-Aldrich, St. Louis, MO, USA) supplemented with 5% fetal bovine serum (FBS), 1% penicillin/streptomycin, were seeded into 96-well plates (Thermo Fisher Scientific, Waltham, MA, USA). Serial dilutions of 10-fold (from 10^−1^ to 10^−8^) were performed in the culture medium. These dilutions were placed onto the TMPRSS2/Vero cells in triplicate and incubated at 37 °C for 96 h. Cytopathic effect was evaluated under a microscope and TCID50/mL was calculated using the Reed–Muench method [50].

### 3.9. Antibacterial Tests

The antibacterial activity was studied by the agar disk diffusion tests [51,52]. Thus, lawns of methicillin-resistant *Staphylococcus aureus* (MRSA), COL [53] and methicillin-resistant *Staphylococcus epidermidis* (MRSE), RP62A [54], in a concentration of approximately 1.5 × 10^8^ colony forming units per mL (CFU/mL) in tryptic soy broth, were cultivated on trypticase soy agar plates. The lawns of bacteria were incubated aerobically at 37 °C for 24 h with the sterilized disks placed upon them. The antibacterial disks showed an inhibition zone (or *halo*) that can be normalized using Equation (1) [51].

(2) $$nw_{halo}=\frac{\frac{d_{iz}\;-\;d}{2}}{d}$$

The term *nw_halo_* represents the normalized width of the antibacterial inhibition zone, *d_iz_* is the inhibition zone diameter and *d* indicates the material disk diameter. The material disk diameter was measured by image software analysis (Image J, Wayne Rasband (NIH), USA). The antibacterial tests were performed three times during two different days (n = 6) to ensure reproducible results.

### 3.10. Antimicrobial Durability of the BAK Coating to Water

The antiviral and antibacterial tests were performed again after washing 1 cm disks of the PET/BAK composite material (BAK Plastic) by immersion in 100 mL of distilled water during 1 min at 24 ± 1 °C to analyze the antimicrobial durability of the BAK coating to water.

### 3.11. Statistical Analysis

The statistical analyses were performed by ANOVA followed by Tukey’s post hoc test (* *p >* 0.05, *** *p >* 0.001) using the GraphPad Prism software (GraphPad Prism 6, GraphPad Software Inc., San Diego, CA, USA).

## 4. Conclusions

A single-use antimicrobial face shield has been developed as the next generation of face protective equipment capable of inactivating enveloped viruses such as phage phi 6 and SARS-CoV-2 after 1 min of contact and multidrug-resistant bacteria such as MRSA and MRSE. This antimicrobial composite material was fabricated by a low-cost procedure consisting of dip-coating of polyethylene terephthalate with benzalkonium chloride. The formation of the antimicrobial coating was demonstrated by atomic force microscopy and field emission scanning electron microscopy with elemental analysis. This composite material avoids viral and bacterial inhalation and entry into the body through the respiratory tract or by splashing (in a surgical operation for example), providing an extra biosafety due to its capacity of inactivating the infectious microorganisms as soon as they are in contact with the protective element. Furthermore, this antimicrobial material is recyclable, and it reduces the generation of infectious biological waste. This antimicrobial material can be used for the fabrication of other face protective equipment such as goggles, helmets, plastic masks and space separation counter or vehicles screens and thus are very promising for the current and future microbial menaces. Nonetheless, further research is needed to contribute significantly on the up-scaling of this technology.

## 5. Patents

Facial protection element against risks of exposure to infectious biological agents (Utility model). U202130782. 15 April 2021.

## Figures and Tables

**Table 1:** Reduction of infection titers in plaque-forming units per mL (PFU/mL) determined by the double-layer assay for the phage phi 6. Logarithm of plaque-forming units per mL (log (PFU/mL)) of the control, untreated PET (U Plastic), PET treated by dip coating with the ethanol-based solvent (S Plastic) and PET with the biofunctional BAK coating (BAK Plastic) at 1 min of viral contact.

Sample	Phi 6 at 1 min (PFU/mL)
Control	4.36 × 10^6^ ± 2.92 × 10^5^
U Plastic	4.38 × 10^6^ ± 1.98 × 10^5^
S Plastic	4.23 × 10^6^ ± 1.36 × 10^6^
BAK Plastic	0.00 ± 0.00

## Data Availability

Data is contained within the article.
